# Podophyllotoxin Extracted from* Juniperus sabina* Fruit Inhibits Rat Sperm Maturation and Fertility by Promoting Epididymal Epithelial Cell Apoptosis

**DOI:** 10.1155/2017/6958982

**Published:** 2017-07-04

**Authors:** Shuwu Xie, Guoting Li, Lijuan Qu, Ruihua Zhong, Ping Chen, Zhigang Lu, Jieyun Zhou, Xiangjie Guo, Zhao Li, Aying Ma, Yueying Qian, Yan Zhu

**Affiliations:** ^1^Laboratory of Reproductive Pharmacology, Key Lab of Reproduction Regulation of NPFPC, SIPPR, IRD, Fudan University, Shanghai, China; ^2^Department of Laboratory Medicine, Shanghai Eighth People's Hospital, Shanghai, China; ^3^Department of Chemistry, Zhejiang University, Hangzhou, Zhejiang, China; ^4^The First Clinical Medical College, Nanjing University of Chinese Medicine, Nanjing, Jiangsu, China; ^5^Technical Institute of Physics and Chemistry, Chinese Academy of Sciences, Beijing, China

## Abstract

This study aimed to investigate the antifertility effect of* Juniperus sabina* fruit on male rats and its possible mechanism, and hence it might be developed as a potential nonhormonal male contraceptive. Male rats were intragastrically fed for consecutive 8-week and 4-week recovery with the fruit of* J. Sabina*, and sperm maturation, serum testosterone level, and histopathology were analyzed. Epididymal epithelial cell culture was prepared for detection of podophyllotoxin activities. Furthermore, cell proliferation, transmission electron microscopy, Annexin V/Propidium iodide, TUNEL, RT-PCR, ELISA, and western blotting were examined. The results showed that rat sperm motility and fertility were remarkably declined after feeding the fruit. Moreover, the fruit targeted the epididymis rather than the testis. After 4-week recovery, more than half of the male rats resumed normal fertility. It was found that podophyllotoxin significantly inhibited epididymal epithelial cell proliferation, promoted cell apoptosis, and increased the mRNA and protein levels of TNF-*α* and the expression levels of cytochrome c, caspase-8, caspase-9, and caspase-3. Our findings suggest that the fruit of* J. sabina* could inhibit male rat sperm maturation and fertility. The potential mechanism might be related to podophyllotoxin, inducing epididymal epithelial cell apoptosis through TNF-*α* and caspase signaling pathway.

## 1. Introduction

With the continuous increase in the world population, more families require birth control. Both husband and wife need to share the responsibility of birth control. A wide variety of oral contraceptives are available in the market for women; however, no contraceptive drug is available for men.

Great advances have been made in hormonal and nonhormonal male contraceptives in the last few decades [1–4]. Hormone-based male contraceptive methods suppress the hypothalamic-pituitary-gonadal axis and thus spermatogenesis. This leads to markedly decreased sperm counts and infertility. Testosterone undecanoate has been investigated in a multicenter phase III clinical trial [5]. However, hormone-based male contraceptives were found to have some undesired side effects such as inducing obesity, hypertension, emotional changes, and increasing the incidence of cancer [6, 7]. Nonhormonal contraceptive agents aim at disrupting spermatogenesis or the sperm-egg interactions by interfering with sperm motility or processes involved in fertilization. Gossypol was one of the most promising nonhormonal male contraceptives, but it has the side effect of hypokalemia paralysis. This led to the termination of the studies on gossypol [8, 9]. So, it is necessary to develop novel nonhormonal male contraceptive methods.

**Figure 1:**
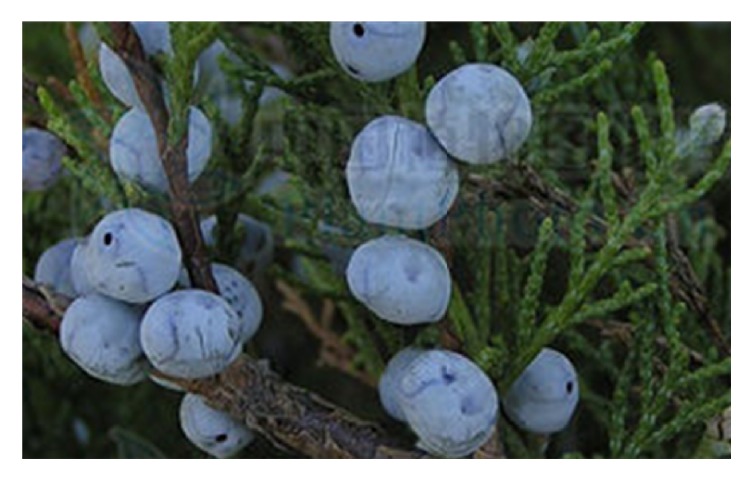
The* Juniperus sabina* from Toli County, Xinjiang Uygur Autonomous Region, China.

*Juniperus sabina* (Cupressaceae, Figure 1) is a species of juniper native to the poor and dry areas. It is a traditional medicinal plant grown mainly in northwestern China. The plant is a trailing dwarf shrub with purple blue fruits growing between 1100 and 2800 m in the dry regions of northwest China. The shrub is up to 1.5–3 m tall, with 5 to 10 mm long needle-like leaves and berry-like fruits. The leaf has been used as an abortifacient agent for women since ancient times [10]. The fruit of* J. sabina* has also served as a folk remedy for male shepherd dog's birth control in northwest China for decades. We have visited the Xinjiang Kazakh ghetto since 2000, with deep understanding of the role of fruit in male dog contraception. The local residents had used it to shepherd reversible contraception for more than one hundred years, but no one performed the relevant pharmacological studies specifically on the antifertility system of animals or human. We were very interested in this discovery and have picked up local fruit of* J. Sabina* to carry out a series of toxicological and pharmacological studies on rats and dogs. The results have shown that the fruit and its extracts had the potential to inhibit male rat or dog fertility, having low toxicity and reversibility [11, 12]. Based on the above studies, we have applied for 2 invention patents in China and the patents have been accepted [11, 12]. It gives us more confidence that the fruit of* J. sabina* and its extracts might be developed as a novel antifertility drug and applied in male contraception.

**Figure 2:**
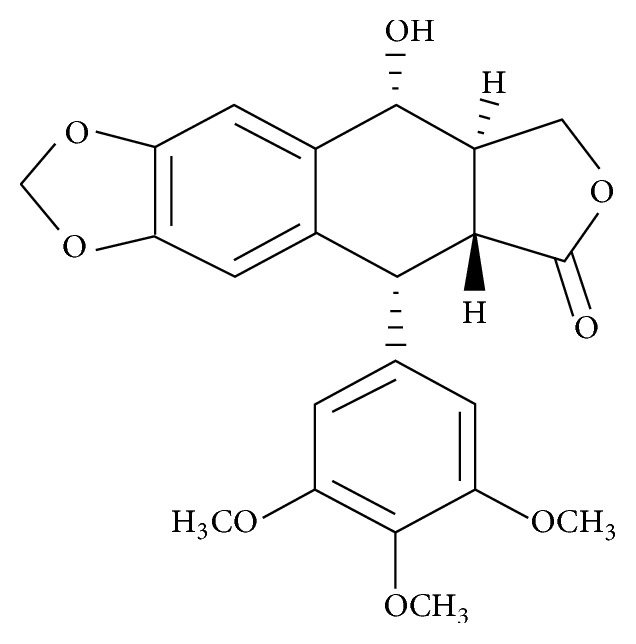
Structure of podophyllotoxin.

Podophyllotoxin (Figure 2), one of the main active components of* J. sabina* fruit, which belongs to lignin, is distributed in various plants including* Podophyllum* [13],* Dysosma* [14],* Diphylleia* [15], and* Juniperus* [16]. Podophyllotoxin was used as a folk remedy for its emetic, cathartic, and anthelmintic effects in the ancient times [17]. Podophyllotoxin has antiviral activity, including human papilloma virus (HPV) and human immunodeficiency virus (HIV) [18]. Actually, podophyllotoxin topical solution (podofilox) and podophyllotoxin tincture (Wartosin) have been clinically applied in the treatment of condyloma acuminatum caused by human papilloma virus (HPV) and other venereal and perianal warts [19], mainly through inhibiting the growth of epithelial cell infected by HPV in epidermis [20]. This chemical has also been reported to inhibit the growth of tumor cells, including cervical carcinoma [21], breast cancer [22], and prostate cancer [14]. Although podophyllotoxin displays antiviral activities in genital warts on male reproductive organs and apoptotic activity in various cancer cells, there has not been evidence showing a relationship between podophyllotoxin induced apoptosis and the effect on normal cells of reproductive organs or male contraception.

In this current study, we further evaluated the antifertility effect of* J. sabina* fruit on male rats in vivo and investigated the possible mechanisms of action of podophyllotoxin on rat epididymal epithelial cells in vitro, and hence they might be developed as potential nonhormonal male contraceptives.

## 2. Materials and Methods

### 2.1. Animals

Forty-eight mature male and 96 virgin female Sprague–Dawley rats (200–250 g) and 15 immature male rats (160–180 g) were purchased from Sino-British Sippr/BK Lab Animal Co. Ltd. (Shanghai, China) for in vivo and in vitro studies, respectively. Male and female rats were maintained on a 12 h light/dark cycle at 20–22°C. Food and water were provided ad libitum. Animal care and all experimental procedures were carried out with the approval of the ethical committee of Shanghai Institute of Planned Parenthood Research (Permit Number: 2013-12).

### 2.2. Collection and Identification of* Juniperus sabina* Fruits

The fruits of* J. sabina* (Cupressaceae) were collected with ultra-hypothermia liquid nitrogen storage in the month of September 2013, from Toli County, Xinjiang Uygur Autonomous Region, China (Figure 1). The collection coordinates were 45°54′59′′N, 84°07′14′′E, and the elevation was 1473 m. The tree was identified by Qian, and its identity was confirmed by W. C. Wang (Academician, Plant Taxonomist from Institute of Botany, Chinese Academy of Sciences). Voucher specimens with collection number Qian et al. 12920 have been deposited in herbaria at Technical Institute of Physics and Chemistry (Chinese Academy of Sciences) and at the Xinjiang Kuitun New World Chemical Co. Limited, China.

### 2.3. Processing and Production of Fruit Powder of* Juniperus sabina*

**Figure 3:**
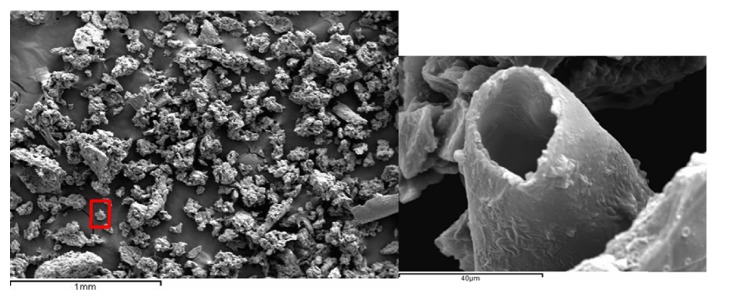
The fruit powder (FP) observation under scanning electron microscope. They were passed through the 200-mesh screen and sieved targeted FP with a diameter of 50–70 *μ*m.

The fruits of* J. sabina* were smashed into fruit powder (FP) in liquid nitrogen at –196°C using Cryogenic Sample Crusher (M100, Beijing Xingshilihe Technology Development Co., Ltd., Beijing, China). The fruit powder was passed through the 200-mesh screen and sieved targeted FP with a diameter of 50–70 *μ*m (Figure 3). The solvent consisted of soy lecithin and corn oil. The FP was dissolved in the solvent (mass ratio 3 : 1) and administered to male rats.

### 2.4. Other Reagents

Dulbecco's modified Eagle's medium/Ham's F-12 (DMEM/F12, 11330), fetal bovine serum (FBS, 12676), and 0.25% trypsin (25200-056) were purchased from Life Technologies (NY, USA). Testosterone was a kind gift from Beijing Zizhu Pharmaceutical Company (Beijing, China). A cell counting kit-8 (CCK-8, CK04) was purchased from Dojindo Molecular Technologies, Inc. (Tokyo, Japan). Fluorescein isothiocyanate (FITC) Annexin V apoptosis detection kit and propidium iodide (PI)/RNase staining buffer (550825) were purchased from BD Biosciences (CA, USA). An In Situ Cell Death Detection Kit (fluorescein, 11684795910) was purchased from Roche (Mannheim, Germany). Rabbit anti-rat *β*-actin (ab8227), cytochrome c (ab53056), caspase-3 (ab47131), caspase-8 (ab25901), and caspase-9 polyclonal antibodies (ab2013) were purchased from Abcam Inc. (Cambridge, MA, USA). Prestained protein ladder (26616) and Halt protease inhibitor cocktail (186229) were purchased from Thermo Scientific (IL, USA). A tumor necrosis factor alpha (TNF-*α*) enzyme-linked immunosorbent assay (ELISA) kit (RTA00) was purchased from R&D Systems Inc. (MN, USA). All other chemicals were purchased from either Sigma-Aldrich (MO, USA) or Beyotime Institute of Biotechnology (Nantong, Jiangsu, China).

### 2.5. Animal Treatment

Forty-eight adult male rats were randomly divided into three groups. Animals in group 1 were treated with solvent 2 g/(kg·d) as the control. Animals in group 2 were treated with 0.8 g/(kg·d) FP. Animals in group 3 were treated with 1.25 g/(kg·d) FP. Each group comprised 16 rats. In each group, half of the rats were used to investigate the antifertility efficacy, and the other half were used to investigate the recovery of infertility after withdrawal of FP treatment. All the animals were given the powdered via gavage once a day for 8 weeks consecutively and weighed weekly. The administration dosages were adjusted according to rat's weight. After discontinuation and mating, half of the animals were anesthetized and dissected, and the remaining recovered for 4 weeks.

### 2.6. Fertility Assay

At 8-week treatment and 4-week recovery, the fertility of each male rat was assessed by naturally mating with two proestrus virgin females. The presence of spermatozoa in vaginal swabs of cohabitated females was regarded as the evidence of successful mating. After 12 days, the number of pregnant (*N*_pregnant_) and nonpregnant (*N*_non-pregnant_) female rats was analyzed. Then, the male fertility rate was calculated as follows:

(1) Fertility  rate%=NpregnantNpregnant+Nnon-pregnant×100%.

### 2.7. Histopathological Study

After mating, the male rats were sacrificed, and the testis and left epididymis were fixed in 4% paraformaldehyde–0.1 M phosphate-buffered saline (PBS) (pH 7.4). Then, the tissue was embedded in paraffin, cut into 5 *μ*m longitudinal sections, and stained with hematoxylin-eosin (HE) for light microscopic examination.

### 2.8. Sperm Motility and Morphological Analysis

Sperm samples were collected from the distal cauda of the right epididymis and used for computer-assisted sperm analysis on the HTM-IVOS using version 12 of the Toxicology Software (Hamilton-Thorne Research, MA, USA). Approximately 5000 cauda epididymal sperms were analyzed for motility in each group. As for morphological analysis, approximately 200–300 sperms per sample were analyzed, and abnormal morphological parameters were broken sperms (head only, tail only, and other breakages) and angulated sperms (bent at midpiece or looped). Then, the frequency of these abnormalities was calculated. The methods were described in a previous study [23].

### 2.9. Detection of the Level of Testosterone in Serum

After the 8-week treatment, serum samples were collected to evaluate the level of testosterone using an ELISA kit, and the optical density (OD) was measured at 450 nm using a microplate reader (Bio-Tek Synergy 2, VT, USA).

### 2.10. Isolation and Culture of Rat Epididymal Epithelial Cells

Epididymal epithelial cell culture was prepared according to the method previously described [24]. Briefly, 15 immature male rats were killed via ether inhalation and the epididymis was removed, dissected free of fat and connective tissues under sterile conditions in Hanks' balanced salt solution (HBSS). Whole epididymis and epididymal segments were dissected out, minced into small fragments, and transferred to 0.25% trypsin (per milliliter of trypsin solution contains 5 mg tissue). After incubation at 32°C for 15 min in a thermobath shaker (100 cycles/min), the sample was centrifuged at low speed (800*g*, 5 min). Then, the supernatant was discarded, and the pellet was suspended in collagenase I in HBSS (1 mg/mL). After incubation at 32°C for 20 min in a thermobath shaker and centrifugation, the sample was allowed to settle for 5 min. Then, the supernatant was discarded, and the sediment, consisting mainly of epididymal epithelial cell aggregates and traces of the collagenase solution, was suspended in DMEM/F12, supplemented with 1 nmol/L testosterone and 10% FBS in a humidified atmosphere (37°C, 5% CO_2_).

### 2.11. Cell Proliferation Analysis

The extracts of the fruit of* J. sabina* were podophyllotoxin (98.5% purity), flavones glycosides, and the essential oil. They were dissolved in dimethyl sulfoxide (DMSO). The cells were digested with 0.25% trypsin at room temperature, and single cell suspension was plated in a 96-well plate in 0.1 mL volumes of the DMEM/F12 medium with 10% FBS and 1 nmol/L testosterone, at a density of 7 × 10^3^ cells per well. After cell attachment, the culture medium of the cells was renewed, and the fruit extracts at the gradient concentrations (0.0001, 0.001, 0.01, 0.1, 1, and 10 *μ*g/mL) were added to the culture medium for appropriate time periods. The final concentration of DMSO in the medium was 0.1%, and the control cells were exposed to an equivalent volume of DMSO. Cell viability was evaluated by adding the CCK-8 solution (10 *μ*L per well) and incubated for 2.5–3 h. The OD was measured at 450 nm using a microplate reader (Bio-Tek Synergy 2).

The inhibition rate of cell proliferation was calculated as follows:

(2) $$\begin{array}{c} \text{Inhibition rate}\left(\;\%\;\right)=\left(\;1-\frac{{OD}_{treated}}{{OD}_{control}}\;\right)\times 100\%. \end{array}$$

The drug concentration required to inhibit cell growth by 50% (IC_50_) was determined by interpolation from dose-response curves. All assays were carried out in quadruplicate.

### 2.12. Transmission Electron Microscopic Observation

Morphological changes in the epididymal epithelial cells were visualized by electron microscopy. Cells treated with DMSO or graded concentrations (0.01, 0.1, and 1 *μ*g/mL) of podophyllotoxin for 48 h were collected by digesting with 0.25% trypsin and sequentially treated with 2.5% glutaraldehyde, 1% osmium tetroxide, propylene oxide, and 1 : 1 Epon-propylene oxide mix. Ultrathin sections were cut and stained with 1% uranyl acetate and lead citrate and then examined under a transmission electron microscope (Philips, CM-120, Tokyo, Japan).

### 2.13. Annexin V/Propidium Iodide Analysis

The epididymal epithelial cells were seeded into 6-well culture plates, and the cells were cultured for 24 h at 37°C in a humidified incubator with 5% CO_2_, followed by a treatment with different concentrations of podophyllotoxin (0.0001, 0.001, 0.01, 0.1, and 1 *μ*g/mL). After 48 h of different treatments, all cells were washed with ice-cold HBSS twice and resuspended in binding buffer at a concentration of 1 × 10^6^ cells/mL. Finally, 5 *μ*L of Annexin V-FITC and 5 *μ*L of PI were added to the sample. After 15 min of incubation at room temperature in the dark, 400 *μ*L of 1x binding buffer was added to each tube. Apoptosis was examined using flow cytometry (Beckman Coulter, CA, USA). The percentage of apoptotic cells was calculated using the internal software system of the Summit 4.3 (Beckman Coulter, CA, USA).

### 2.14. Terminal Deoxynucleotidyl Transferase-Mediated dUTP-Fluorescein Nick-End Labeling Assay

After incubation with different concentrations of podophyllotoxin for 48 h, the cells were rinsed three times with PBS and fixed with 4% paraformaldehyde. Based on the terminal deoxynucleotidyl transferase-mediated dUTP-fluorescein nick-end labeling (TUNEL) kit protocol, the cells were permeabilized with 0.1% Triton X-100 in 0.1% sodium citrate for 2 min on ice and then rinsed twice with PBS. The DNA nick-end labeling reaction was performed using 50 *μ*L of TUNEL reaction mixtures, including 45 *μ*L of enzyme solution and 5 *μ*L of nucleotide mix at 37°C for 60 min. Then, the samples were rinsed three times with PBS and analyzed under a fluorescence microscope (DM3000, Leica, Germany) with an excitation wavelength in the range of 450–500 nm and detection wavelength in the range of 515–565 nm. The apoptotic percentage of each sample was denoted by normalizing the apoptotic cell amount in three equal areas to the total cell amount in the corresponding areas (Facscalibur Flow Cytometer, Becton, Dickinson and Company, NJ, USA).

### 2.15. Quantitative Real-Time Reverse Transcription-Polymerase Chain Reaction

TNF-*α* was measured by real-time reverse transcription-polymerase chain reaction (RT-PCR). Total RNAs were extracted from epididymal epithelial cells (treated with graded concentrations of podophyllotoxin for 48 h) by adding 1 mL of TRIzol Reagent (Invitrogen, CA, USA) and reverse-transcribed to form cDNAs using the ABI7500 Real-Time PCR system (Applied Biosystems, CA, USA) according to the manufacturer's instructions. Glyceraldehyde-3-phosphate dehydrogenase (GAPDH) was used as an endogenous quantity control [25]. The primer sequences used in the quantitative real-time PCR reactions were as follows: GAPDH, forward: 5′-AGTGCCAGCCTCGTCTCATAG-3′, reverse: 5′-CGTTGAACTTGCCGTGGGTAG-3′; TNF-*α*, forward: 5′-CACCACGCTCTTCTGTCTACTG-3′, reverse: 5′-TCCGCTTGGTGGTTTGC-3′. The PCR products of GAPDH and TNF-*α* were 192 and 174 bp, respectively. The total volume of 20 *μ*L PCR reactions was prepared by mixing 1.0 *μ*L of cDNA sample, 10.0 *μ*L of 2x SYBR Premix Ex Taq II (Tli RNaseH Plus, Takara Bio Inc., Shiga, Japan), 0.4 *μ*L of forward primer (10 *μ*M), 0.4 *μ*L of reverse primer (10 *μ*M), and 8.2 *μ*L of ddH_2_O. The real-time PCR reactions were performed on a Rotor-Gene 3000 instrument (Corbett Research, Mortlake, Australia). The final results were presented as the ratios of the relative amount of the target gene (TNF-*α*) to the control gene (GAPDH) using the 2^–ΔΔCT^ equation, where ΔΔCT = CT  (target  gene) − CT (internal control gene), according to the method described by Livak and Schmittgen [26]. The results were analyzed using the Rotor-Gene 6.0 software (Corbett Research). The results were comparable to those normalized to GAPDH, and the statistical analysis was performed using the average relative mRNA levels from three independent samples.

### 2.16. ELISA for Detection of the Levels of TNF-*α*

The cells were treated with gradient concentrations of podophyllotoxin for 48 h, and cell culture supernatants were collected. The level of TNF-*α* in culture supernatants was measured by ELISA according to the manufacturer's instructions.

### 2.17. Western Blot Analysis

The cells (2 × 10^5^/mL; 10 mL) were planted in a culture flask (75 cm^2^), cultured for 24 h, and subsequently treated with gradient concentrations of podophyllotoxin for 48 h. Cytoplasm extracts were prepared with 300 *μ*L of cell lysis buffer on ice for 30 min and then centrifuged at 10,000*g* at 4°C for 10 min, and the supernatant was collected. The protein concentration was quantified using the bicinchoninic acid protein assay kit (Beyotime Institute of Biotechnology). Proteins were mixed with sodium dodecyl sulfate polyacrylamide gel electrophoresis sample loading buffer. In total, a 40 *μ*g sample of protein was separated on a 12.5% polyacrylamide gel and blotted onto a polyvinylidene fluoride membrane. The blots were blocked with 5% skimmed milk for 2 h at room temperature and incubated with anti-*β*-actin (1 : 4000), anti-cytochrome c (1 : 1000), anti-caspase-8 (1 : 2000), anti-caspase-9 (1 : 2000), and anti-caspase-3 (1 : 2000) antibodies for 12 h at 4°C. Subsequently, the polyvinylidene fluoride membranes were washed with Tris-buffered saline with Tween 20 (TBST) buffer three times and incubated with goat anti-rabbit antibody in blocking buffer for 2 h at room temperature. Then, the polyvinylidene fluoride membranes were washed with TBST buffer three times and detected with enhanced chemiluminescence (Beyotime Institute of Biotechnology). The bands were then visualized using the Gel Doc XR+ system and quantified using the ChemiDoc XR+ imaging system (Bio-Rad, CA, USA).

### 2.18. Statistical Analysis

All statistical analyses were performed using the Statistical Package for the Social Sciences (SPSS, Version 20.0; IL, USA). The continuity correction of Pearson's chi-square test was used to analyze the male rat fertility rates between FP treatment and control groups. The one-way analysis of variance test with post hoc multiple comparisons by the least significant difference method was used for comparison between groups. Differences were considered statistically significant at *P* < 0.05. Data are presented as mean ± standard deviation (SD).

## 3. Results

### 3.1. Effects of the Fruit of* Juniperus sabina* on Sperm Motility and Morphology

**Figure 4:**
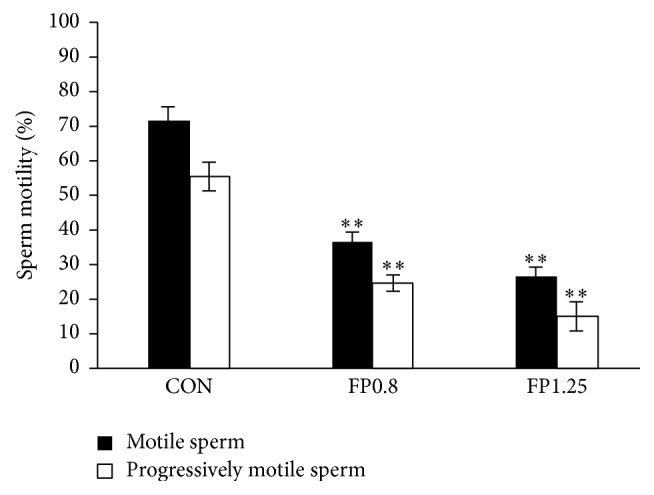
Male sperm motility and progressive motility after 8-week treatment. Male rats were treated with the solvent (CON) or with the fruit powder [FP: 0.8 and 1.25 g/(kg·d)]. Data are presented as percentages. ^*∗∗*^*P* < 0.01 versus the control group.

A significant decrease in the percentage of motile and progressively motile sperms from the caudal epididymis of rats was observed in* Juniperus sabina* fruit powder- (FP-) treated groups compared with the control group (*P* < 0.01, Figure 4). Meanwhile, FP treatment also caused a marked decrease in other sperm motility parameters (Table 1).

**Figure 5:**
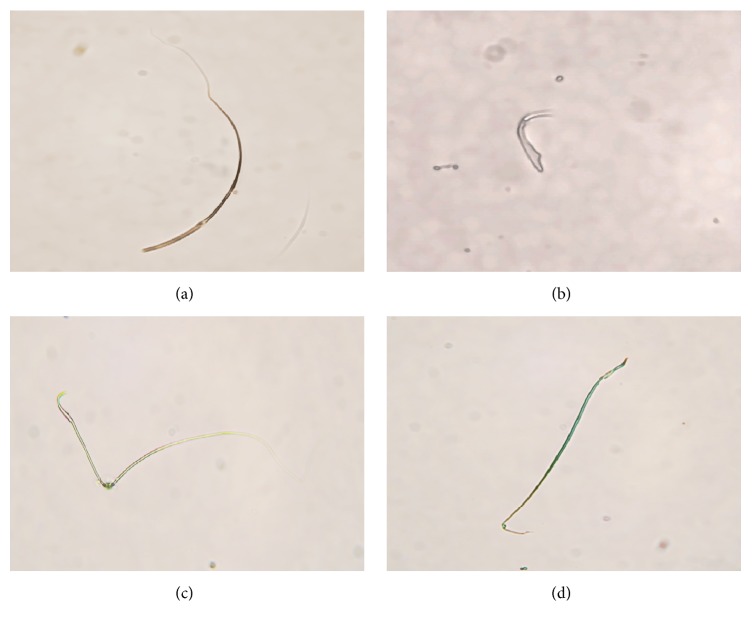
Structure of rat epididymal teratosperms under a phase contrast microscope. (a) Headless sperm (×200). (b) Tailless sperm (×400). (c) Angulated sperm (×200). (d) Broken sperm (×200).

**Figure 6:**
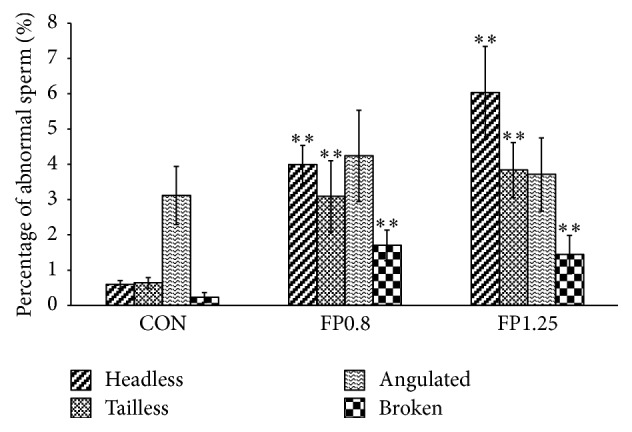
Percentages of abnormal sperms after 8-week treatment. Data are presented as percentages. ^*∗∗*^*P* < 0.01 versus the control group.

As for sperm morphology, the abnormal percentages of tailless, headless, and broken sperms also increased significantly with FP treatment (*P* < 0.01, Figures 5 and 6).

### 3.2. Effects of the Fruit of* Juniperus sabina* on the Fertility in Male Rats

**Figure 7:**
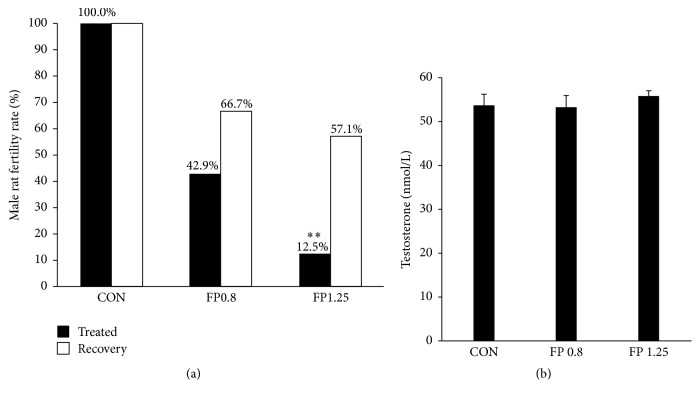
(a) Male fertility rates after 8-week treatment and 4-week recovery. Data are presented as percentages. ^*∗∗*^*P* < 0.01 versus the control group. (b) The level of serum testosterone in the experimental rats was measured by ELISA. No significant difference was found between groups (*n* = 8).

After 8-week treatment, the results showed that the fertility of rats in 1.25 g/(kg·d) FP-treated group significantly decreased compared with that in the control group (*P* < 0.01). All female rats mating with male rats in the control group were pregnant, and therefore the male rat fertility rate was 100%. However, the male rat fertility rates were 42.9% and 12.5% in the 0.8 g/(kg·d) and 1.25 g/(kg·d) FP-treated groups, respectively (Figure 7(a)).

After 4-week recovery, the percentages of rats that resumed normal fertility were 67.0% and 57.0% in the 0.8 and 1.25 g/(kg·d) FP-treated groups, respectively; no significant difference in the recovery fertility rate was found between the control and the FP-treated groups (Figure 7(a)).

### 3.3. Serum Testosterone Analysis

The level of serum testosterone in the experimental rats is shown in Figure 7(b). No significant changes in the testosterone level were found among all the groups (*P* > 0.05, Figure 7(b)).

### 3.4. Effect of* Juniperus sabina* on Histopathological Changes in Testis and Epididymis

**Figure 8:**
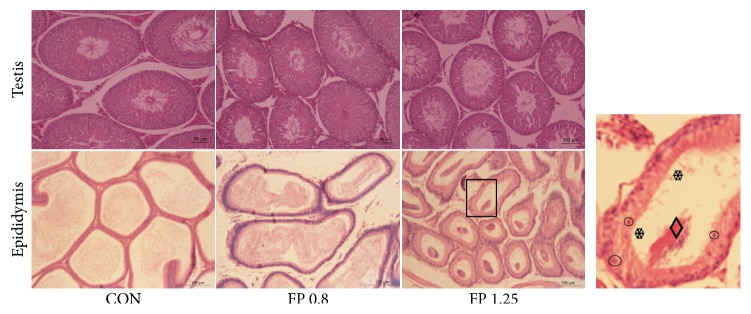
Analysis of testis and epididymis micrographs. Male rats were treated with the solvent (CON) or with* Juniperus sabina* fruit powder [FP: 0.8 and 1.25 g/(kg·d)]. *❄* (snowflake): short and sparse stereocilia; ◊ (rhombus): decreased spermatozoa and spermatid clustering in the epididymal lumen; ○ (circle): epithelial cell dislodgement from the epithelium.

As shown in Figure 8, normal histopathology was observed in the testis and epididymis of the control group. The testis of the control group presented a clear structure of seminiferous tubules and an orderly arrangement of spermatogenic cells. The testes of the FP-treated rats were similar to those of the control group. A large number of sperms were found in the testicular seminiferous tubule of both the control and the FP-treated rats.

The epididymis in the control group was composed of numerous ductus epididymis filled with spermatozoa and marginal stereocilia. In the 0.8 g/(kg·d) FP-treated group, the gaps between the ductus epididymis significantly increased without evident abnormal pathological changes. However, in the 1.25 g/(kg·d) FP-treated group, lots of abnormal exfoliated spermatozoa aggregated and the ductus epididymis became significantly atrophied with marginal stereocilia desquamating and epididymal epithelium damage.

### 3.5. Effects of* Juniperus sabina* Fruit Extracts on the Growth of Rat Epididymal Epithelial Cells

**Figure 9:**
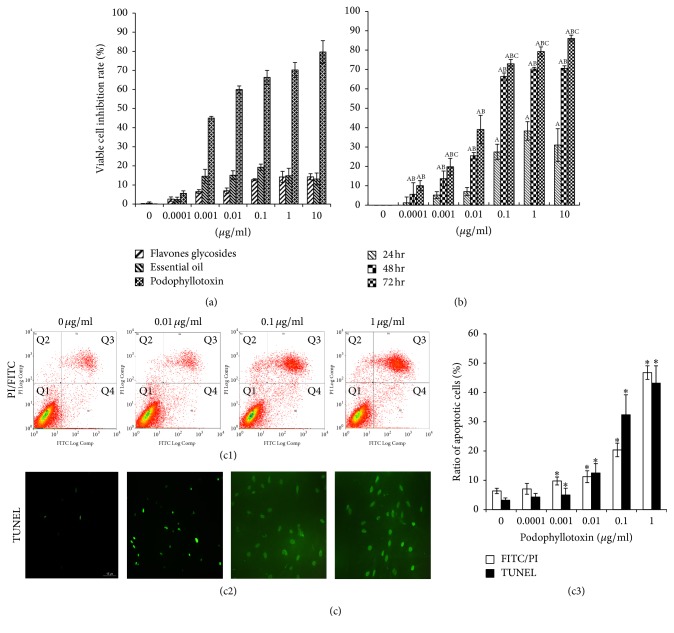
(a) Cell inhibition rates of podophyllotoxin, flavone glycosides, and the essential oil on rat epididymal epithelial cells. The cells were treated with the extracts for 48 h and measured using the CCK-8 method. (b) Cell inhibition rates of podophyllotoxin on rat epididymal epithelial cells at 24, 48, and 72 h, as assessed by the CCK-8 method. Epididymal epithelial cells were treated with graded concentrations of podophyllotoxin (0–10 *μ*g/mL). Values represent mean ± SD (*n* = 4). ^a^*P* < 0.05, compared with the control (0 *μ*g/mL); ^b^*P* < 0.05, compared with the 24 h treated cells; ^c^*P* < 0.05, compared with the 48-h treated cells. (c) Podophyllotoxin induced apoptosis in rat epididymal epithelial cells. The cells were treated with podophyllotoxin for 48 h. (c1) Representative micrographs of cell apoptosis analyzed by flow cytometry (Q1: normal; Q2: necrosis; Q3: late apoptosis; Q4: early apoptosis). (c2) Representative micrographs of cell apoptosis detected by TUNEL (green fluorescence represents apoptotic cells). (c3) Percentage of apoptotic cells was quantified using the Summit software of flow cytometry and TUNEL-positive cells under a microscope. Data are represented as mean ± SD (*n* = 3);  ^*∗*^*P* < 0.05 versus the control group.

As shown in Figure 9(a), podophyllotoxin remarkably suppressed the viability of rat epididymal epithelial cells; however, flavones glycosides and the essential oil had no significant effect on the growth of epididymal epithelial cells. Therefore, the time- and dose-dependent effect of podophyllotoxin on cell viability was further studied. The results revealed that podophyllotoxin exhibited an inhibitory effect on the proliferation of epididymal epithelial cells in a time- and dose-dependent manner (Figure 9(b)). The cell inhibition rates of the 48 and 72 h treated groups exhibited statistically significant differences compared with the control group (*P* < 0.05). The 50% inhibitory concentration (IC_50_) values were 24.68  ±  13.25, 0.0124  ±  0.0003, and 0.0062 ± 0.0025 *μ*g/mL at 24, 48, and 72 h, respectively.

### 3.6. Effect of Podophyllotoxin on the Apoptosis of Epididymal Epithelial Cells

The flow cytometry results revealed that podophyllotoxin induced increment of nonviable PI-positive and Annexin V-positive cells, which were in the apoptotic phase. The apoptotic cell scatters appeared gradually in a dose-dependent manner (Figure 9(c)). The apoptotic rates represented the sum of the late and the early apoptotic rates, which were 6.37 ± 0.9%, 7.07 ± 1.85%, 9.77 ± 1.36%, 11.23 ± 2.01%, 20.37 ± 2.32%, and 46.77 ± 2.32% in the 0, 0.0001, 0.001, 0.01, 0.1, and 1 *μ*g/mL podophyllotoxin groups, respectively (Figure 9(c1)).

The cell apoptosis was further assayed using the TUNEL method. As shown in Figure 9(c2), much more TUNEL-positive cells (green fluorescence represented apoptotic cells) were detected by labeling DNA single-strand breaks with dUTP-fluorescein at the 3′-OH ends of the nuclei, compared with the control group. The apoptotic percentage in the control group was 3.2%  ±  0.78%. However, the number of apoptotic cells increased significantly in a dose-dependent manner after treatment with 0.0001–1 *μ*g/mL of podophyllotoxin for 48 h, and the apoptotic percentage increased from 4.31%  ±  1.22% to 5.03%  ±  2.32%, 12.54%  ±  3.22%, 32.46%  ±  6.79%, and 43.24%  ±  5.91%, respectively (Figure 9(c3)).

### 3.7. Podophyllotoxin Induced Ultrastructural Changes in Epididymal Epithelial Cells

**Figure 10:**
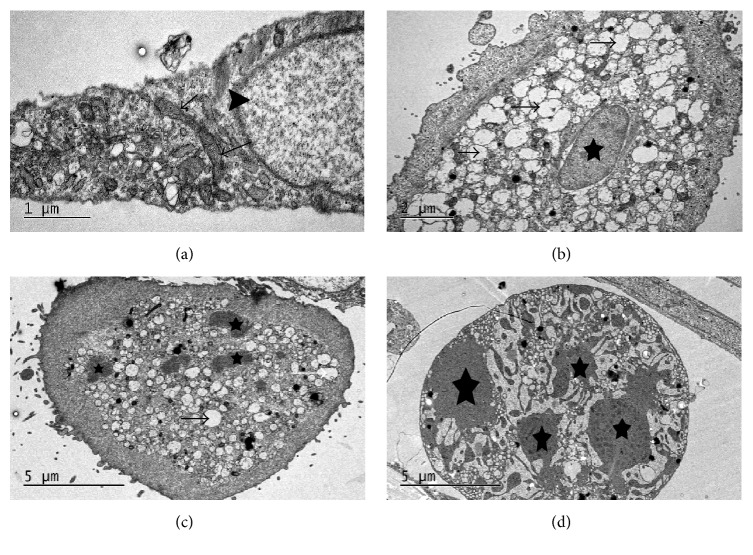
Ultrastructural changes in epididymal epithelial cells. The cells were cultured in gradient concentrations of podophyllotoxin for 48 h. (a) 0 *μ*g/mL podophyllotoxin: normal mitochondria (arrow) and cell nucleus (arrow head). (b) 0.01 *μ*g/mL podophyllotoxin: mitochondrial vacuolation (arrow) and nucleus shrinkage (star). (c) 0.1 *μ*g/mL podophyllotoxin: vacuolation (arrow) and nuclear fragmentation (star). (d) 1 *μ*g/mL podophyllotoxin: nuclear condensation and fragmentation (star).

The epididymal epithelial cells in the blank control group were found to present a normal ultrastructure such as mitochondria and microtubules (Figure 10(a)). However, epididymal epithelial cells treated with 0.01 *μ*g/mL podophyllotoxin for 48 h exhibited early apoptotic-like ultrastructural characteristics, such as mitochondrial vacuolation, cytoplasmic disorganization, and cell volume shrinkage (Figure 10(b)). Cells treated with 0.1 *μ*g/mL podophyllotoxin after 48 h exhibited middle-stage apoptotic-like ultrastructural characteristics of chromatin condensation and fragmentation (Figure 10(c)). Cells treated with 1 *μ*g/mL podophyllotoxin after 48 h exhibited ultrastructural characteristics of late apoptotic cells such as structural disorganization and disaggregation of cell nucleus (Figure 10(d)).

### 3.8. Podophyllotoxin Enhanced mRNA Level of TNF-*α*

**Figure 11:**
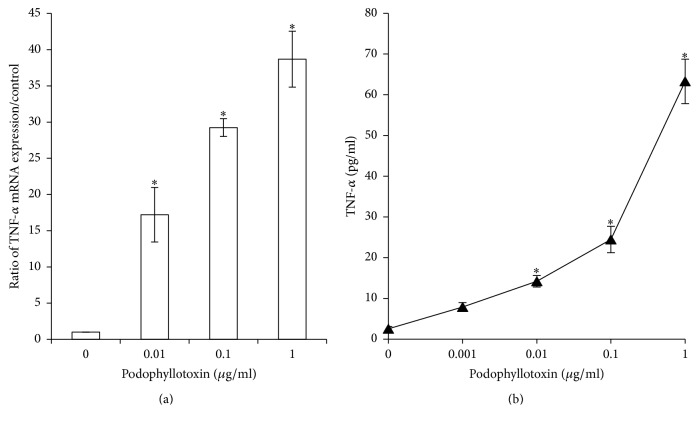
(a) Effects of podophyllotoxin on the mRNA level of TNF-*α* in rat epididymal epithelial cells, as assessed by real-time RT-PCR. The cells were treated with graded concentrations of podophyllotoxin (0, 0.01, 0.1, and 1 *μ*g/mL) for 48 h. Data are presented as mean ± SD of three independent samples. ^*∗*^*P* < 0.05 versus the control cells. (b) Effects of podophyllotoxin on TNF-*α* secretion of rat epididymal epithelial cells, as assessed by ELISA. Cells were treated by graded concentrations of podophyllotoxin (0, 0.001, 0.01, 0.1, and 1 *μ*g/mL) for 48 h. Data are presented as mean ± SD of three independent samples. ^*∗*^*P* < 0.05 versus the control group.

RT-PCR analysis for TNF-*α* was performed to validate the alteration in gene expression. Compared with the control group, a concentration-dependent increase in the mRNA level of TNF-*α* was observed after treatment with podophyllotoxin at concentrations of 0.01 (17-fold), 0.1 (29-fold), and 1 (38-fold) *μ*g/mL (*P* < 0.05, Figure 11(a)). This result indicated that the expression of TNF-*α* was enhanced in the podophyllotoxin-treated epididymal epithelial cells.

### 3.9. Podophyllotoxin Increased the Secretion of TNF-*α*

The level of TNF-*α* in cell culture supernatants was assayed after the cells were treated with podophyllotoxin for 48 h. The results obtained by ELISA showed that podophyllotoxin increased the level of TNF-*α* in the culture supernatants in a dose-dependent manner, and a pronounced increase was observed in cells treated with 1 *μ*g/mL compared with the control group (Figure 11(b)).

### 3.10. Podophyllotoxin Upregulated the Levels of Caspase-8, Cytochrome c, Caspase-9, and Caspase-3 Proteins

**Figure 12:**
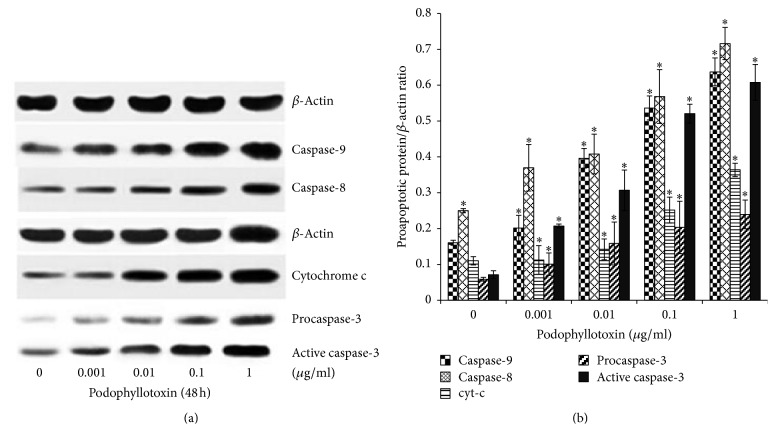
(a) Representative blot and (b) quantitative results demonstrating the effect of podophyllotoxin on apoptosis-related proteins in rat epididymal epithelial cells. The cells were treated with graded concentrations of podophyllotoxin (0, 0.001, 0.01, 0.1, and 1 *μ*g/mL) for 48 h. Data are presented as mean ± SD of three independent samples. ^*∗*^*P* < 0.05 versus the control group.

Western blotting was used to determine changes in several proapoptotic proteins. As low as 0.001 *μ*g/mL of podophyllotoxin could increase the level of caspase-9 in a dose-dependent manner. Compared with the control group, a concentration-dependent increase in the level of caspase-8 was found, and pronounced increments were observed at the concentrations of 0.01 *μ*g/mL and 0.1 *μ*g/mL podophyllotoxin. Furthermore, a significant increase (*P* < 0.05) in the protein level of cytochrome c was noted after treatment with 1 *μ*g/mL podophyllotoxin (Figure 12). Along with the activation of caspase-8, cytochrome c, and caspase-9, both procaspase-3 (32 kDa) and active caspase-3 (17 kDa) gradually increased and the level of active caspase-3 was more than that of procaspase-3 treated with 0.1 *μ*g/mL podophyllotoxin, indicating that caspase-3 was activated by podophyllotoxin at concentrations greater than or equal to 0.1 *μ*g/mL.

## 4. Discussion

This novel study found that the fruit of* J. sabina* has the ability to suppress sperm maturation and fertility of male rats in vivo, and its effect seemed to be reversible after withdrawal. Furthermore, it was also found that the target of* J. sabina* fruit was the epididymis rather than the testis. Finally, the potential mechanism of action might be related to podophyllotoxin, one of its active ingredients, inducing epididymal epithelial cell apoptosis through TNF-*α* and caspase signaling pathway.

Epididymis plays a key role in sperm maturation because sperms remain immature and lack the ability to move and fertilizing as they leave the testis [27]. The highly convoluted epididymal duct provides an appropriate environment for spermatozoa. The role of epididymal secretions is to maintain sperm vitality, permit the development of sperm motility, and protect them against noxious agents [28]. This specialized luminal environment is conducive to the maturation process through the absorptive and secretory activities of the epididymal epithelium [29]. When the morphology and function of epididymal epithelial cells are abnormal, spermatozoa are immature and infertile [30]. Moreover, the epididymis could be viewed as an ideal target for developing male contraceptives because spermatozoa reside for a relatively short time in the epididymis, and no genetic transformation is known to occur as spermatozoa undergo epididymal maturation [29].

Many phytochemicals are found to have contraceptive efficacy, including gossypol, a phenolic compound derived from cottonseed and triptolide, and the extract of* Tripterygium wilfordii* [10]. Gossypol was reported to inhibit male fertility by targeting both epididymis and testis [31, 32], and triptolide damages mainly the epididymal spermatozoa [33, 34]. Unfortunately, both of them were found to result in serious side effects and irreversible infertility [35, 36].

This study found that oral treatment with 0.8 and 1.25 g/(kg·d)* J. sabina* fruit for consecutive 56 days significantly inhibited sperm motility and declined fertility. In particular, the fertility of the animals in the 1.25 g/(kg·d) group decreased to 12.5% without affecting the testosterone level. Four weeks after the treatment was withdrawn, the fertility of more than 50% male rats was recovered to the normal level. Histopathologically, all FP-treated rats suffered impairment on epididymis rather than testis. The most prominent defects shown in FP-treated rats were ductus epididymal atrophy and immature sperm aggregation in the ductus epididymis. Testis appeared to be unaffected and exhibited no histological defects. These results suggested that* J. sabina* fruit damaged the epididymal epithelium and caused the aggregation of the epididymis sperms at the given doses. Different from gossypol acetate, which inhibits male fertility via targeting both testis and epididymis [32],* J. sabina* fruit targets selectively the epididymis rather than acting on the testis, and this may be one of the reasons that attribute to the reversal of antifertility effects.

The influence of three different extracts of the* J. sabina* fruit on the growth of epididymal epithelial cells was further analyzed in vitro, and it was found that one of the extracts, podophyllotoxin, significantly inhibited the proliferation of epididymal epithelial cells. Accordingly, it was presumed that podophyllotoxin was likely to be the major factor that reduced the male fertility. However, no evidence on the inhibitory effect of podophyllotoxin on epididymis is available to date. Therefore, this study next investigated the effect of podophyllotoxin on cultured epididymal epithelial cells and found that podophyllotoxin induced cell apoptosis remarkably.

Apoptosis, known as programmed cell death, is a strict controlled and energy-dependent process of cell death. Activation of apoptosis results in a cascade of characteristic biochemical events, which change cellular morphology and induce death [37]. Cells undergoing apoptosis display some relatively well-characterized morphological changes including phosphatidylserine externalization [38], cytochrome c leakage from the mitochondria, caspase activation, cell and nuclear volume reduction, chromatin condensation, and DNA damage [39]. The present study provided evidence supporting the effectiveness of podophyllotoxin in inhibiting the growth and inducing the apoptosis of rat epididymal epithelial cells. The significant increase in the apoptotic rate in podophyllotoxin-treated cells via the annexin V/PI analysis suggested that podophyllotoxin induced apoptosis via phosphatidylserine externalization on the cytoplasmic surface of the cell membrane. As characteristic hallmarks of apoptosis, mitochondrial vacuolation and DNA fragmentation of the nucleus were also observed in podophyllotoxin-treated cells using transmission electron microscopy and TUNEL assay. These results indicated that podophyllotoxin inhibited the growth of epididymal epithelial cells, the mechanism more likely to be involved in the activation of apoptosis.

Many pathways are known to regulate cell apoptosis [40–42]. The expression level of TNF-*α* and cytochrome c increased in vitro after the treatment with podophyllotoxin in the present study. Also, the expression levels of caspase-8, caspase-9, and caspase-3 were affected by TNF-*α* and cytochrome c [43]. It was confirmed that TNF-*α* and cytochrome c could activate caspase-8 and caspase-9, respectively, and both caspase-8 and caspase-9 activated caspase-3 finally [44]. Caspase-3 is one of the most important caspases in the process of apoptosis. Once activated, caspase-3 cleaves numerous cellular proteins related to DNA degradation, cycle regulation, and DNA repair [43]. A previous study demonstrated that podophyllotoxin could facilitate the release of cytochrome c from the mitochondrion into the cytoplasm [45]. Furthermore, the increased expression levels of caspase-8, caspase-9, and caspase-3 were also observed, indicating that podophyllotoxin could induce apoptosis by promoting mitochondrial permeabilization and activate the upstream caspase to release cytochrome c from the mitochondrion into the cytoplasm. These findings confirmed that the apoptosis-inducing effect of podophyllotoxin in rat epididymal epithelial cells was mediated through the TNF-*α* and mitochondrial signaling pathway, similar to previous studies [45, 46]. The results of this in vitro study provided further evidence that the epididymis was indeed a possible target organ for the fruit of* J. sabina*.

## 5. Conclusions

**Figure 13:**
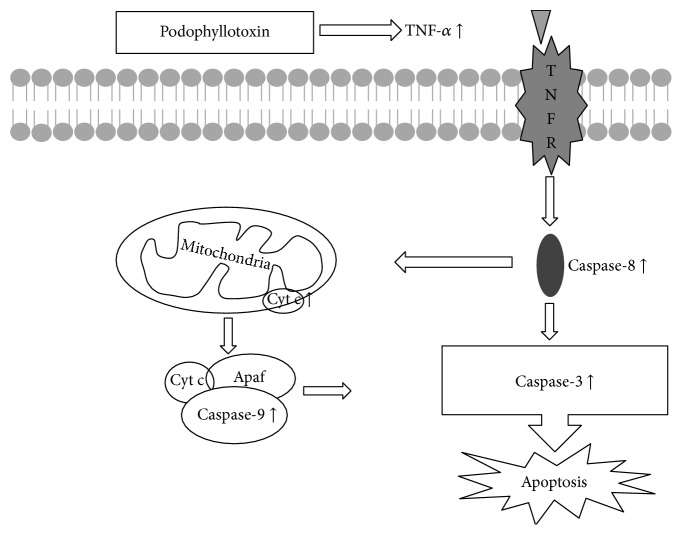
Summary of the possible mechanisms of podophyllotoxin induced apoptosis in rat epididymal epithelial cells. Podophyllotoxin increases the mRNA and secretion level of TNF-*α*, and TNF-*α* activates caspase-8 indirectly. Caspase-8 can activate caspase-3 and cleave Bid, leading to cytochrome c release and caspase-9 activation. Both caspase-8 and caspase-9 then activate executioner caspase-3, which orchestrates the removal of cells by programmed cell death.

This study demonstrated that the fruit of* J. sabina* had an ability of inhibiting sperm maturation and fertility of SD male rats in vivo, especially targeting the epididymis, which might contribute to reversing the antifertility effect after the FP treatment was withdrawn. The mechanism of action is likely related to podophyllotoxin, one of the fruit extracts, which inhibits the growth of epididymal epithelial cells and promotes apoptosis via the TNF-*α* and caspase signaling pathways (Figure 13). Further animal studies are necessary to determine the efficacy and safety of the extracts of* J. sabina* fruit for its antifertility effect.

## Figures and Tables

**Table 1:** Effect of FP treatment on other sperm motion parameters (mean ± SD).

Groups	VAP (*μ*m/s)	VSL (*μ*m/s)	VCL (*μ*m/s)	ALH (*μ*m)	BCF (Hz)	STR (%)	LIN (%)
CON	149.2 ± 10.6	109.2 ± 10.2	231.4 ± 15.8	15.3 ± 1.3	27.1 ± 2.1	70.2 ± 4.1	49.2 ± 4.0
FP0.8	123.2 ± 5.9^*∗∗*^	70.9 ± 6.8^*∗∗*^	235.2 ± 16.5	12.5 ± 1.3^*∗∗*^	22.1 ± 1.1^*∗∗*^	58.0 ± 6.2^*∗∗*^	35.2 ± 3.4^*∗∗*^
FP1.25	118.4 ± 7.2^*∗∗*^	66.4 ± 5.7^*∗∗*^	233.6 ± 21.9	11.4 ± 1.2^*∗∗*^	20.2 ± 1.2^*∗∗*^	48.8 ± 2.4^*∗∗*^	26.1 ± 3.5^*∗∗*^

^*∗∗*^ *P* < 0.01 versus the control group. ALH, amplitude of lateral head displacement; BCF, beat cross frequency; LIN, linearity; STR, straightness; VAP, average path velocity; VCL, curvilinear velocity; VSL, straight-line velocity.

## Abbreviations

TNF-*α*: Tumor necrosis factor alpha
FP: Fruit powder
CASA: Computer-assisted sperm analysis
VAP: Average path velocity
VCL: Curvilinear velocity
VSL: Straight-line velocity
ALH: Amplitude of lateral head displacement
BCF: Beat cross frequency
TUNEL: Terminal deoxynucleotidyl transferase-mediated dUTP-fluorescein nick-end labeling.
